# An improved sequential auction with complementarity for pricing the construction land quota

**DOI:** 10.1371/journal.pone.0241397

**Published:** 2020-10-29

**Authors:** Jingyu Liu, Weidong Meng, Yuyu Li, Bo Huang

**Affiliations:** 1 School of Economics and Business Administration, Chongqing University, Chongqing, China; 2 School of Economics and Management, Chongqing Normal University, Chongqing, China; Institute for Advanced Sustainability Studies, GERMANY

## Abstract

The paradox between idle homesteads in rural areas and the shortage of construction land in urban areas is concerning. Hence, local governments encourage farmers to reclaim their idle homesteads and farmlands to generate quota. However, the current quota price, which is based on the land reclamation cost, is often inadequate to motivate farmers. This study proposed that the construction land quota and construction land are complementary items, and hence, their pricing should be studied simultaneously instead of independently. Therefore, the classical sequential auction model with complementarity was improved using the core characteristics of quota transactions and those were applied to design optimal pricing mechanisms. Subsequently, the influence of relevant factors on the bidding price was analysed. The results indicated that the bidding price decreases with an increase in the number of bidders and that a bidder’s revenue is at a loss when they acquire the quota but fail to obtain the land; hence, the revenue probability is lost. However, bidding prices increase when the fine of delaying construction increases. To raise the quota price and encourage relatively more farmers to reclaim their idle homesteads, local governments should appropriately increase the delaying construction fines or repurchase the quota at a premium from the bidder who acquires the quota but fails to obtain the land, which is beneficial for easing conflict between construction land demands and farmland protection and for promoting the sustainable development of China’s social economy.

## Introduction

Since the implementation of the Open and Reform Policy of 1978 in China, urbanisation has occurred at an unprecedented rate, increasing from 17.88% in 1978 to 59.58% in 2018 [1]. The increasing population and economic activities within cities led to a demand for more space for housing, industrial parks, facilities and services, and infrastructure. This rapid urbanisation has created enormous pressure on land supply, sustaining urban expansion nationwide, and farmland protection [2, 3]. According to the China Land and Resources Statistical Yearbook, approximately 230 thousand hectares of farmland was converted into construction land between 2003 and 2014 [4]. In response to the pressure of farmland protection, while simultaneously providing space for urbanisation, Chinese government has implemented annual long-term plans to impose a strict control over the quota allocated to local governments, for converting the farmland into construction use land [5, 6]. However, with an increasing demand for the construction land, this system resulted in the highly severe ‘shortages’ of construction land supply [7, 8].

Meanwhile, with the rapid urbanisation, rural workforces migrated to cities, leading to a trend of population decline in rural areas, which decreased from 790 million in 1978 to 590 million in 2016 [9]. Due to China’s dualistic household registration system, urban residents are prohibited from purchasing rural homesteads. Consequently, withdrawing or reusing idle rural homesteads caused by rural population outflow has become difficult [10]. This phenomenon led to a large waste of land resources and low land-use efficiency.

To alleviate this problem, certain local governments in China, such as Chengdu and Chongqing, undertook the lead for the innovation of construction land quota generation policies [11]. Subsequently, other local governments in China, such as Guangzhou, Nanjing, and Zhejiang, established the same or similar mechanisms. Under this mechanism, local governments encourage farmers to reclaim their idle homesteads as farmland and generate additional quotas [12]. Thereafter, local governments can sell these quotas to developers for urban construction, and the total amount of farmland remains unchanged; the farmers who lost their lands receive a payment according to the bidding price of developers who acquire the said quota [13]. However, in practicing quota transactions, the current price of the quota is set based on the cost of reclaiming idle homesteads, which does not reflect the true intrinsic value of construction land quotas and cannot stimulate farmers to reclaim their idle homesteads [14]. Thus, for the Chinese government, designing a reasonable pricing mechanism of quotas that reflects its true value and encourages more farmers to reclaim their idle homesteads is critical to achieve highly sustainable land use and development in China.

Studies on the transactions of construction land quotas have focused on two core fields. The first field is the general introduction of quota trading, including the significance, processes, and basic characteristics of quota transactions. Liu and Long [15] analysed the core characteristics of the sources and transactions of land quotas and indicated that governments should strengthen the interprovincial transaction of quotas in the future. Tan [16] found that meeting supplier demands by using reclamation costs as the compensation of construction land quotas was difficult, and the price should consider land development rights. Tan [17] described the transaction process of construction land quotas and claimed that the intrinsic value of the land quota should be further explored. Li et al. [18] concluded that quota implementation showed how the core goals of land use—ensuring food security and easing urban sprawl—had met expectations. Task implementation showed that the utilisation of built-up lands was considerably improved. Fang and Tian [19] emphasised that the quota system might serve as an effective tool for China to combat undesirable growth patterns in the future. Zhou et al. [20] analysed mechanisms and paths that lay behind land consolidation to promote poverty alleviation and explained successful practices through a case study.

The second field is the analysis of intrinsic values, transaction prices, and income allocation of quotas. Zhang and Wang [21] and Feng et al. [22] have concluded that so far, the price mechanisms of construction land quotas does not truly reflect the intrinsic value of the land quotas by analysing quota trading in Chongqing. Yang et al. [23] reported that the establishment of a pricing system of the construction land quota should emphasise on two aspects: the transfer of land development rights and regional development through the support of the pricing system. Hao and Du [24] considered that the essence of quota transactions was in the transfer of land development rights. Qiu and Qiu [25] reported that in the current cost-oriented pricing of the quota, the price of the land quota comprises the generation cost of the construction land quota (the majority being the reclamation cost of homesteads) and the compensatory cost of land development rights. Hu and Wang [26] stated that the quota price of construction lands should comprise the land reclamation cost, quota trading cost, compensation price of land development rights, and risk income. However, it only comprises transaction and reclamation costs in the current pricing mechanism. Hu and Tan [27] and Meng and Xiong [28] have proposed that the government should reduce its impact on price in quota transactions and encourage moderate competitions among bidders and that suppliers and demanders should finalise construction land quota prices.

However, these papers have not studied the method of designing a feasible pricing mechanism to set reasonable prices for the quota, which should completely reflect the intrinsic value of the quota. When prices are reasonable, only then many farmers will be motivated to supply quotas [29, 30]. The construction land quota enables developers to establish their projects in advance so that the intrinsic value of the quota is in the surplus profits of completing the projects in advance. This indicates that ‘the quota of the construction land’ and ‘the construction land’ are complementary items. Because these extra profits are developers’ private information, the optimal approach to reveal them is through the auction mechanism. Hence, a two-stage sequential auction with complementarity between the quota and construction land is effective to price the quota.

Some studies have investigated the sequential auction of complementary items. Subramaniam and Venkates [31] found that separate auctions of two complementary items were superior to the auction of bundled items, when at least four bidders were present. Menezes [32] studied two sequential second-price auctions with positive and negative synergies and found that the existence of positive and negative synergies implied the decrease and increase in expected prices, respectively. Sørensen [33] studied the sequential auction equilibrium prices of complementary items, which were randomly equivalent, and analysed the relationship between the expected equilibrium bidding price and valuation probability. Martin and Norman [34] assumed that a seller sold two items to independent potential buyers, examined the nature of sellers’ optimal mechanism for externalities, and found that the optimal mechanism could be implemented through an indirect mechanism that essentially charges the winner for the externality value. Donna and Espin–Sanchez [35] found that when two items were complementary, one bidder can win both items by paying a high price for the first item, thereby deterring others from bidding on the second item. Wang et al. [36] introduced quantity discount into sequential second sealed-bid price auctions with two stochastically equivalent complementary items and found that adopting quantity discounts can induce intense competition among bidders and increase sellers’ ex-ante expected surplus. Corazzini et al. [37] experimentally studied sequential procurement auctions and discovered that overall behaviours during the second auction were consistent with sequential rationality and that the first auction bids decreased with the bidders’ capacity. From the aforementioned studies, the sequential auction with complementarity has been deeply studied and already used in reality. For example, the use of railways in the European Union is split into several segments and periods of being complementary, which are auctioned sequentially [38]. Although the sequential auction with complementarity has not been applied to the transaction of construction land quotas, it is a reasonable and feasible method to price the quota.

This paper makes three important contributions. First, it improves the classical sequential auction model with complementarity on the basis of the main characteristics of quota transactions. In classical sequential auctions with complementarity, winning during the first stage increases the item value in the second stage. In quota transactions, failing during the first stage decreases the item value in the second stage. Second, this paper suggests that construction land quotas and construction lands should be studied simultaneously as complementary items, rather than separately and proposes that the main function of land quotas is to ensure construction lands are developed in advance and the quota value is the addition of the time value in construction land use. The intrinsic quota value provides extra profit for developers and is their private information. Thus, our improved sequential auction model with complementarity is effective to price quotas and construction lands. Third, this paper studies the influence of relevant factors on quota bidding prices through theoretical and numerical analyses. The model and conclusions of this paper can guide governments to form a scientific and effective pricing policy to reflect the true value of quotas, and hence, improve the efficiency of construction land use. In addition, they are applicable to the allocation and use of limited resources with constraints.

The remaining sections of this paper are organised as follows. Section 2 introduces the problem and variables. Section 3 analyses bidders’ and farmers’ equilibrium solutions. Section 4 presents the numerical analysis to further explore sequential auction strategies and discussion. Section 5 summarises and highlights the core features of the price mechanism of land quotas based on the proposed sequential auction.

## Problem and variables

### Problem

In China, the construction land quota, predominantly allocated from the central government, is essential for local governments for developing farmlands for construction purposes [39]. When the quota of one year completes, continuing the construction is illegal. To solve this problem, the local government must generate an extra quota. Chengdu and Chongqing are pilot practices that are recently followed by many other provinces, including Hunan, Hubei, Zhejiang, Jiangsu, Anhui, Guangdong, and Shandong. In this practice, when local governments plan to develop construction lands without a quota from the central government, they encourage farmers to reclaim their idle homesteads voluntarily to obtain an extra quota and sell it to developers in need. Therefore, the relationship between lands and quotas is typically complementary. To set a reasonable quota price that completely reflects the intrinsic value of the quota and motivates farmers, the local government organises a two-stage sequential auction to sell the quota and construction land. The quota and construction land are sold during the first and second stage, respectively.

Several developers plan to develop their projects on construction lands. For this purpose, they must win both the construction land and quota. In practice, the developer who wins both can begin to establish their project immediately after the auction. The developer who wins only the construction land must wait until the quota is won in the future.

After the first stage, farmers who provide the quota by reclaiming their idle rural homesteads obtain a single payment of 85% of the quota price.

**Fig 1:**
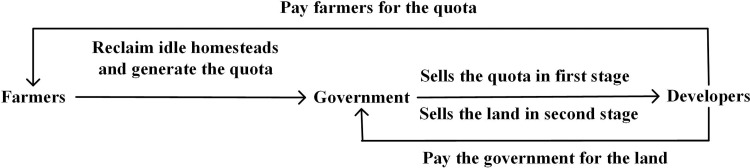
Relations among the government, developers, and farmers.

Fig 1 illustrates the relations among the government, developers, and farmers.

Without the loss of generality, we made the following assumptions. First, the local governments and developers are risk neutral. Second, the developers participating in the first and second stage auctions are the same. Third, the rule of the second price sealed-bid auction is applied to both the stages, that is, the bidder with the highest price wins the quota or construction land but is only required to pay for the second highest price of auction. Finally, all information, except the developers’ valuations of the quota and construction land, is common knowledge.

### Variables

Local governments hold a two-stage sequential auction to sell a quota and piece of construction lands, which are sold in the first and second stages, respectively. In both the stages, bids are submitted simultaneously, and the bidders are required to only pay for the second highest price of the auction when they win the quota or construction land.

*n*(≥2) risk-neutral bidders participate in both the first and second stages of the auction. Those bidders’ valuations of the quota (‘item 1’) and construction land (‘item 2’) are *V*_1*i*_ and *V*_2*i*_, respectively, *i* =1,2,∙∙∙,*n* respectively. *V*_1*i*_ and *V*_2*i*_ follow the uniform distribution of [0, *a*] and [0, 1], respectively [40, 41]. In the second stage, the bidder who successfully wins the quota is referred to as *w*, and *l* refers to the other *n* − 1 bidders who lose the first auction.

However, according to core transaction characteristics of the quota, the bidder who wins both the quota and construction land can start their project immediately after the auction. The bidder who wins the construction land but fails to obtain the quota must wait until they win the quota in the future. During this period, the bidder must pay the fine for delaying construction. The fine (or penalty cost) reduces the bidder’s profit of developing the construction land [42–44]. Consequently, losing the quota decreases the value of the construction land from *V*_2*i*_ to *V*_2*i*_/*R*, *i* =1,2,∙∙∙,*n*. *R* can be expressed as the parameter of land value depreciation. *R* relates to the fine of delaying construction. The higher is the fee, the higher is *R*.

If a bidder acquires the quota but fails to use it within a specified period, they sell back the quota to the local government at the original price. In this case, the bidder suffers the corresponding loss of revenue *γ*_*i*_ (0 < *γ*_*i*_ <1). Before the two-stage auction, bidder *i* (*i* =1,2,∙∙∙,*n*) believes that the probability of them winning the construction land is *ρ*_*i*_. Therefore, during the first stage of the auction, bidder *i* considers this expected loss of (1 − *ρ*_*i*_)*γ*_*i*_ on their bidding price of the quota.

In the first stage of the auction, the equilibrium bidding price of bidders *i* is *β*_1*i*_(·), and *E*_1*i*_, *i* =1,2,∙∙∙,*n*, is the ex-ante expected surplus of bidders *i* or the difference between the expected value of the construction land quota for bidder *i* and the expected price they pay. In the second auction stage, the equilibrium bidding price of bidders *i* is *β*_2*i*_(·). If a bidder *i* wins the quota, its ex-ante expected surplus is $E_{2i}^{w}$, *i* =1,2,∙∙∙,*n*. If bidder *i* fails to win the quota, its ex-ante expected surplus is $E_{2i}^{l}$, *i* =1,2,∙∙∙,*n*. Among them, the difference in the ex-ante expected surplus between the winner and losers is Δ. Δ is referred to as the added surplus and denoted as $\Delta =E_{2i}^{w}-E_{2i}^{l}$, *i* =1,2,∙∙∙,*n*. It indicates that the bidder who wins the quota of the construction land can avoid the fine on delaying construction, that is, they can obtain an extra profit.

## Equilibrium solutions

### Bidders’ equilibrium bidding price

We used the backward induction method to obtain the equilibrium bidding price of bidders in the first and second stage. We can obtain Proposition 1 as follows:

#### Proposition 1

In the sequential auction of the construction land quota and construction land, the equilibrium bidding price of bidders in the first and second stage are respectively:

$$\beta_{1i}(V_{1i})=V_{1i}+\Delta -(1-\rho_{i})\gamma_{i},i=1,2,\cdots ,n,$$ (1)

$$\{\begin{array}{c} \begin{array}{ccc} \begin{array}{cc} \begin{array}{cc} \beta_{2i}^{w}(V_{2i})=V_{2i} & \end{array} & i=1,2,\cdots ,n \end{array} & & \end{array}\\ \begin{array}{cc} \beta_{2j}^{l}(V_{2j})=V_{2j}/R & \begin{array}{cc} j=1,2,\cdots ,n & j\neq i \end{array} \end{array} \end{array},$$ (2)

where, Δ = (*R* − 1)[(*R*– 1)*n* + 2]/(2*R*^2^*n*).

Proof: We used the backward induction method to obtain the equilibrium bidding price of all bidders in the first stage.

To characterise the equilibrium behaviour of bidders, we must proceed backwards, starting with the second auction (the construction land auction).

First, according to Menezes et al. [40] and Leufkens and Peeters [45], bidders who fail to obtain the quota during the first stage reduce their valuation of the construction land by *R* times. They also reduce their expected payoff during the second auction. Therefore, we can obtain the equilibrium bidding price of all bidders in the second stage as follows:

$$\{\begin{array}{c} \begin{array}{ccc} \begin{array}{cc} \begin{array}{cc} \beta_{2i}^{w}(V_{2i})=V_{2i} & \end{array} & i=1,2,\cdots ,n \end{array} & & \end{array}\\ \begin{array}{cc} \beta_{2j}^{l}(V_{2j})=V_{2j}/R & \begin{array}{cc} j=1,2,\cdots ,n & j\neq i \end{array} \end{array} \end{array}$$ (3)

When bidder *i*, *i* =1,2,∙∙∙,*n*, wins the construction land quota, the maximum bidding price in the second stage for other *n* − 1 bidders can be defined as *z*/*R*, where *z* represents the largest valuation of the land, except for bidder *i*. The ex-ante expected surplus of bidder *i* in the second action is as follows:

$$\begin{array}{c} E_{2i}^{w}=\iint_{V_{2i}>z/R}\left(V_{2i}-z/R)(n-1\right)z^{n-2}d_{V_{2i}}d_{z}\\ =\int_{0}^{\frac{1}{R}}d_{V_{2i}}\int_{0}^{RV_{2i}}(V_{2i}-z/R)(n-1)z^{n-2}d_{z}\\ +\int_{\frac{1}{R}}^{1}d_{V_{2i}}\int_{0}^{1}(V_{2i}-z/R)(n-1)z^{n-2}d_{z}\\ =1/\left[R^{2}n(n+1)\right]+\left(R-1)(Rn-n+2\right)/\left(2R^{2}n\right) \end{array}$$ (4)

If bidder *i*, *i* =1,2,∙∙∙,*n*, fails during the first stage and bidder *k*, *k* = 1,2,…,*n k* ≠ *i*, wins during the first stage, the bidding price of bidder *k* in the second stage can be defined as *z*_1_. The maximum bidding price of the other *n* − 2 bidders in the second stage, except for *i* and *k*, can be defined as *z*_2_/*R*, where *z*_2_ represents the largest valuation of the land, except for bidder *i* and *k*. Now, the ex-ante expected surplus of bidder *i* in the second stage can be acquired as follows.

$$\begin{array}{l} E_{2i}^{l}={∭}_{}(V_{2i}/R-max\{z_{1},z_{2}/R\})(n-2)z_{1}^{n-3}d_{V_{2i}}d_{z_{1}}d_{z_{2}}\\ \begin{array}{cc} & \text{=} \end{array}\int_{0}^{1}d_{V_{2i}}\int_{0}^{\frac{V_{2i}}{R}}(V_{2i}/R-z_{1})d_{z_{1}}\int_{0}^{Rz_{1}}(n-2)z_{2}^{n-3}d_{z_{2}},\\ \begin{array}{cc} & +\int_{0}^{1}d_{V_{2i}}\int_{0}^{V_{2i}}(n-2)z_{2}^{n-3}d_{z_{2}}\int_{0}^{\frac{z_{2}}{R}}(V_{2i}/R-z_{2}/R)d_{z_{1}} \end{array}\\ \begin{array}{cc} & =1/\left[R^{2}n(n+1)\right] \end{array} \end{array}$$ (5)

where *i* =1,2,∙∙∙,*n* and $\Delta =E_{2i}^{w}-E_{2i}^{l}=\left(R-1)[(R-1)n+2)\right]/\left(2R^{2}n\right)$.

From the discussion, we can obtain the following information: Δ = (*R* − 1)[(*R*– 1)*n* + 2]/(2*R*^2^*n*).

If bidder *i*, *i* =1,2,∙∙∙,*n*, wins the construction land quota, they can obtain an extra profit of Δ, that is, the construction land quota brings an extra value of Δ for bidder *i*. Consequently, the total value, or the bidding price of bidder *i*, is the sum of the original value of the construction land quota and extra value. In addition, it includes the revenue lost because the bidder who acquires the quota fails to obtain the construction land. This can be expressed as follows:

$$\beta_{1i}(V_{1i})=V_{1i}+\Delta -(1-\rho_{i})\gamma_{i},\;\text{where}\;\Delta \text{=}(R-1)[(R-1)n+2)]/\left(2R^{2}n\right).$$

Q.E.D.

From Proposition 1, we found that the bidders’ equilibrium bidding price during the first stage comprises the instinct value of the quota for bidders. Therefore, our two-stage sequential auction is effective and feasible to reveal the true value of the quota and is better than the current method of cost-based pricing adopted by Chongqing and other cities. Therefore, it can motivate many farmers to reclaim their idle homesteads.

Additionally, we found that bidders’ equilibrium bidding prices during the first stage is a function of the number of bidders *n*, the fine of delaying construction *R*, the probability of them winning the construction land *ρ*_*i*_, and the corresponding revenue loss *γ*_*i*_. During the second stage, the equilibrium bidding price of bidder *i* who fails to obtain the quota is the function of the fine of delaying construction *R*. Therefore, we can propose Proposition 2 as follows:

#### Proposition 2

During the first stage, the equilibrium bidding price of the bidders decreased when the number of bidders increased; however, it increased with the increase in the fine of delaying construction. During the second stage, the equilibrium bidding price of the bidders who failed to acquire the construction land quota decreased with the increase in the fine of delaying construction.

Proof: By taking the first derivative of the equilibrium bidding price of bidders during the first stage, with respect to the number of bidders *n*, and the fine of delaying construction *R*, we can obtain *∂β*_1*i*_ (*V*_1*i*_)/*∂n* < 0 and *∂β*_1*i*_ (*V*_1*i*_)/*∂R* > 0, respectively, *i* =1,2,∙∙∙,*n*. Therefore, the equilibrium bidding price of bidders during the first stage is a strictly decreasing and increasing function of the number of bidders *n* and fine of delaying construction *R*, respectively. that is, in the first stage, the equilibrium bidding price of bidders decreases with an increase in the number of bidders *n* and decreases with an increase in the fine of delaying construction *R*.

When we then examined the first derivative of the losers’ equilibrium bidding price during the second stage with respect to the fine of delaying construction *R*, we can get $\partial \beta_{2j}^{l}(V_{2j})/\partial R<0$, *i* =1,2,∙∙∙,*n*. In the second stage, the losers’ equilibrium bidding price is the strictly decreasing function of the fine of delaying construction *R*, that is, the equilibrium bidding price of the losers decreases with the increase in the fine of delaying construction *R*.

Q.E.D.

Proposition 2 shows that the equilibrium bidding price of bidders during the first stage decreases with the increase in the number of bidders mainly because a decrease in the expected surplus revenue of winning the quota. The practical reasons for this phenomenon are as follows: when the number of bidders increases, the competition among them becomes more intense, and the probability of a bidder obtaining the quota decreases. Consequently, the expectation of the extra revenue of obtaining the quota also decreases, and bidders pay less on the quota.

This conclusion is similar to the argument of Sørensen [33] that bidders would bid less aggressively during the first auction because the number of bidders increased. In reality, with the increase in the number of bidders, the competition among bidders becomes more intense. The probability of bidders acquiring both the item and the additional value brought by the item during the first stage also reduce. As a result, bidders decrease the price of the item during the first stage.

Hence, we can conclude from Proposition 2 that the equilibrium bidding price of bidders during the first stage increases with the increase in the parameter of land value depreciation *R*. As aforementioned, land value depreciation is caused by the developer delaying construction because of their failure to obtain the quota. The higher the fine of developers for delaying construction is, the larger *R* is. As a result, with the increase in *R* or the fine, the loss of failure to obtain the quota increases, that is, the expectation of extra revenue for obtaining the quota increases, raising the value and bidding price of the quota.

This finding is consistent with the conclusions drawn by Wang et al. [36] and Leufkens and Peeters [41]. The results showed that *R* positively affects the price of the first item during the first stage. In practice, *R* is positively correlated with the fine of delaying construction. The higher is the fine, the more is the cost saved through the quota.

Finally, we found that during the second stage, the equilibrium bidding price of bidders who fail to obtain the quota decreases with the increase in the fine of delaying construction. The core reasons are similar to the aforementioned reasons. The larger is *R*, the higher is land value depreciation for losers during the first stage. Hence, they are willing to pay less for the land, and the equilibrium bidding price of the land decreases.

#### Proposition 3

In the first stage, the equilibrium bidding price of the quota rises with the increase in the probability of bidders’ belief that they can win the land during the second stage and decreases with the increase in the revenue loss of bidders losing during the second stage.

Proof: By taking the first derivative of the equilibrium bidding price of the quota with respect to probability *ρ*_*i*_ and revenue loss *γ*_*i*_, we obtain *∂β*_1*i*_ (*V*_1*i*_)/*∂ρ*_*i*_ > 0 and *∂β*_1*i*_ (*V*_1*i*_)/*∂γ*_*i*_ < 0, respectively. Thus, the equilibrium bidding price of the quota is the strictly increasing and decreasing function of probability *ρ*_*i*_ and revenue loss *γ*_*i*_, respectively, that is, during the first stage, the equilibrium bidding price rises and decreases with the increase in probability *ρ*_*i*_ revenue loss *γ*_*i*_, respectively.

Q.E.D.

Proposition 3 shows that the equilibrium bidding price of the quota decreases with the increase in the revenue loss of bidders losing in the second stage mainly because with the increase in the revenue loss, bidders’ expectation of revenue loss rises, resulting in the value depreciation of the quota. Hence, bidders decrease their bidding price on the quota.

Additionally, Proposition 3 shows that the equilibrium bidding price of the quota rises with the increase in the probability of bidders’ belief that they can win the land during the second stage. The core reason for this finding is that with the increase in the probability of bidders’ belief of winning the land during the second stage, the probability of the bidder’s belief of suffering revenue loss decreases. This leads to a decrease in their expectation of revenue loss. Hence, bidders raise their bidding price on the quota.

The aforementioned two conclusions are consistent with Lu’s findings [47]. The results show that the revenue loss of bidders losing during the second stage negatively affects the price of the first item during the first stage, and the probability of a bidder’s belief that they can win the land during the second stage is positively correlated with the bidding price during the first stage. Thus, repurchasing the quota at a premium from the bidder is an effective approach to improve the bidding price of the construction land quota and is the optimum method to ensure farmers’ land income.

According to Proposition 3, we can present a political suggestion that the government may repurchase the quota at a premium from the bidder who acquires the quota but fails to obtain the land. Compared with Chongqing’s current practice of repurchasing at the original price, repurchasing at a premium can reduce bidders’ revenue loss and raise their bidding price on the quota.

### Bidders’ ex-ante expected surplus

First, we acquired the ex-ante expected surplus of bidders during the first stage. We can obtain Proposition 4 as follows.

#### Proposition 4

The ex-ante expected surplus of bidders during the first stage is

$$E_{1i}=1/(n^{2}+n),i=1,2,\cdots ,n.$$ (6)

Proof: Except bidder *i*, *i* =1,2,∙∙∙,*n*, the maximum bidding price of the other *n* − 1 bidders during the first stage is denoted as *z*_3_.

$$\begin{array}{l} E_{1i}=\iint_{V_{1i}>z_{3}}\left(V_{1i}-z_{3}\right)(n-1)z_{3}^{n-2}dV_{1i}d_{z_{3}}\\ \begin{array}{cc} & =\int_{0}^{1}dV_{1i}\int_{0}^{V_{1i}}(V_{1i}-z_{3})(n-1)z_{3}^{n-2}d_{z_{3}} \end{array},\\ \begin{array}{cc} & =1/\left(n^{2}+n\right) \end{array} \end{array}$$ (7)

where *i* =1,2,∙∙∙,*n*.

Q.E.D.

Proposition 4 indicates that bidders’ ex-ante expected surplus in the first stage is the function of the number of bidders *n*. Therefore, we can obtain Proposition 5 as follows:

#### Proposition 5

In the first stage, the ex-ante expected surplus of bidders decreases with the increase in the number of bidders.

Proof: By taking the first derivative of bidders’ ex-ante expected surplus with respect to the number of bidders *n*, we can obtain *∂E*_1*i*_/*∂n* < 0, *i* =1,2,3,∙∙∙,*n*. Therefore, during the first stage, the ex-ante expected surplus of bidders is the strictly decreasing function of the number of bidders *n*, that is, the ex-ante expected surplus of bidders decreases with the increase in the number of bidders *n*.

Q.E.D.

Proposition 5 shows that the ex-ante expected surplus of bidders decreases with the increase in the number of bidders. The core reasons are as follows: When more bidders are present, the competition for the construction land quota becomes more intense. The difference between the equilibrium bidding price of bidder *i*, *i* =1,2,3,∙∙∙,*n* (or the expect value of the quota for them) and expected price they pay (or the highest bidding price, which is lower than its price) decreases, that is, bidders’ ex-ante expected surplus decreases.

### Analysis of farmers’ expected income

We analysed the farmers’ expected income and obtained Proposition 6 as follows:

#### Proposition 6

The expected income of farmers decreases with the increase in the number of bidders and revenue loss and increases with the increase in the delaying construction fine and the probability of the bidders’ belief of winning the land during the second stage.

Proof: According to Proposition 2, when the number of bidders and revenue loss increases, the equilibrium bidding price of all bidders, including the second highest price, decreases. Therefore, the farmers’ income (i.e. the second highest price) decreases with the increase in the number of bidders and revenue loss.

Moreover, according to Proposition 2, the equilibrium bidding price of all bidders increases with the rise in the fine of delaying construction and the probability of the bidders’ belief of winning the land during the second stage. Consequently, the income of farmers increases.

Q.E.D.

Proposition 6 shows that the farmers’ expected income decreases with the increase in the number of bidders and revenue loss mainly because of the following reasons: When the number of bidders increases, the competition among them becomes more intense; hence, the probability of a bidder obtaining the quota decreases. Consequently, the expectation of the extra revenue of obtaining the quota decreases. Therefore, bidders pay relatively more for the construction land. With the increase in the revenue loss, the quota value decreases. Consequently, they pay less for the quota, and farmers’ expected income decreases.

Proposition 6 also shows that the expected income of farmers increases with the increase in the fine of delaying construction and the probability of the bidders’ belief of winning the land in the second stage. As aforementioned, the higher is the fine on delaying construction, the higher is the value of the construction land quota and the bidding price of all bidders. Therefore, the expected income of farmers increases. In addition, as probability increases, the bidders bid relatively more for the quota. As a result, the biding price of the quota raises, and the expected income of farmers increases.

This result is indirectly supported by Brendstrup [46]. With the increase in the fine of delaying construction, their bidding price on the quota increases, and consequently, farmers’ income increases. In addition, this finding is the same as that of Lu [47]. The premium repurchase quota can not only effectively show the intrinsic value of the construction land quota and improve bidders’ bidding price of the quota but also assuredly provide the farmers’ expected income.

Therefore, local governments should properly increase the fine of delaying construction or repurchase the quota at a premium from the bidder who acquires the quota but fails to obtain the land. Consequently, the farmers’ income and their motivation of reclaiming idle homesteads increases, and the efficiency of land use improves.

### Numerical analyses

Numerical simulation is conducted through Matlab programming to thoroughly analyse the price mechanism of construction land quota, based on the sequential auction.

Assume that a local government holds a two-stage sequential auction to sell the quota and construction land. After the announcement of the auction, ten bidders participate in bidding. According to the assumption, the bidders valuation of the construction land follows the uniform distribution of [0,1]. According to the relevant data of Chongqing country land exchange, the transaction price of the construction land is considerably lower than that of the construction land, and the price difference between construction land and the quota is approximately ten times (refer to Table 1 for details) [21]. Therefore, we assumed that the upper limit of uniform distribution of the construction land quota *a* = 0.1, that is, the bidders’ valuation of the construction land quota, follows the uniform distribution of [0,0.1]. All bidders’ estimates of the construction land quota and construction land are generated randomly in [0,0.1] and [0,1], according to practices and model assumptions (Table 2). According to the land management law of the People’s Republic of China, the fine for delaying construction is 10%–20% of the land price. Without the loss of generality, parameter *R* is 1.3. In addition, the probability that the bidder obtains the land is generated randomly in [0,1], and the probability in this paper is 0.4. Without the loss of generality, the revenue loss is generated randomly in [0,1], and the revenue loss in this paper is 0.05. We analysed the equilibrium bidding strategy of bidders and the impact of related factors.

**Table 1:** Average price of the quota and construction land in Chongqing from 2011 to 2015 (ten thousand yuan /mu).

Year Price	2011	2012	2013	2014	2015
Quota	24.39	18.70	20.28	18.70	18.67
Construction land	213.03	153.83	197.61	198.08	190.71

Data source: Chongqing country land exchange

**Table 2:** Values of the land quota and construction land of bidders.

Bidder Value	1	2	3	4	5	6	7	8	9	10
*V*_1*i*_	0.09	0.07	0.065	0.075	0.054	0.032	0.082	0.036	0.012	0.041
*V*_2*i*_	0.7	0.5	0.42	0.31	0.87	0.65	0.12	0.6	0.9	0.81

Solving the two-stage auction model of construction land and quota, we can obtain the equilibrium bidding price of bidders during both stages in Table 3.

**Table 3:** Equilibrium bidding price of bidders during both stages.

Bidder Price	1	2	3	4	5	6	7	8	9	10
*Β*_1*i*_(·)	0.13	0.11	0.105	0.115	0.094	0.072	0.122	0.076	0.052	0.081
$\beta_{2i}^{w}(\cdot )or\beta_{2j}^{l}(\cdot )$	0.7	0.38	0.32	0.24	0.67	0.5	0.09	0.46	0.69	0.62

In the first stage, the highest and second highest bidding prices, which are 0.13 and 0.122, respectively, are received from bidder 1 and 7, respectively (Table 3). Therefore, bidder 1 wins the quota and pays 0.122. As a result, the farmers income is 0.104. During the second stage, the highest and second highest bidding prices are 0.7 and 0.69, respectively, from bidder 1 and 9, respectively. Therefore, bidder 1 wins the construction land and pays 0.69.

In the following section of this paper, we present a sensitivity analysis to explore the impact of the parameter of land price depreciation on the equilibrium bidding price of bidders, the bidders’ ex-ante expected surplus and the expected income of farmers.

**Fig 2:**
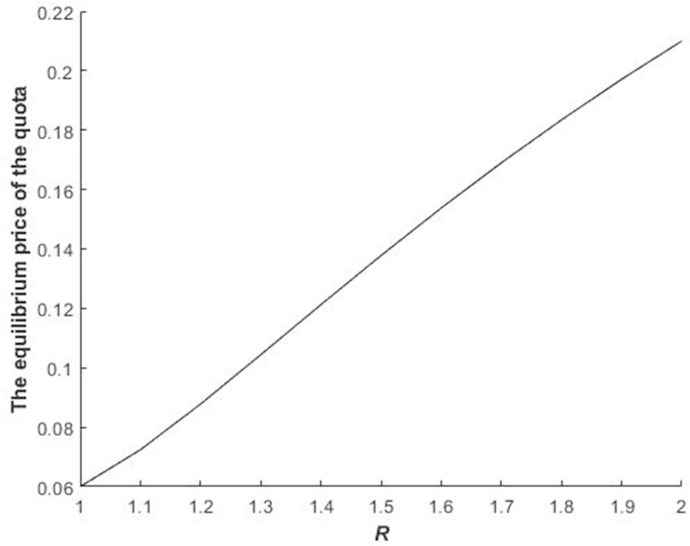
Impact of *R* on the bidders’ equilibrium bidding price of the quota during the first stage.

**Fig 3:**
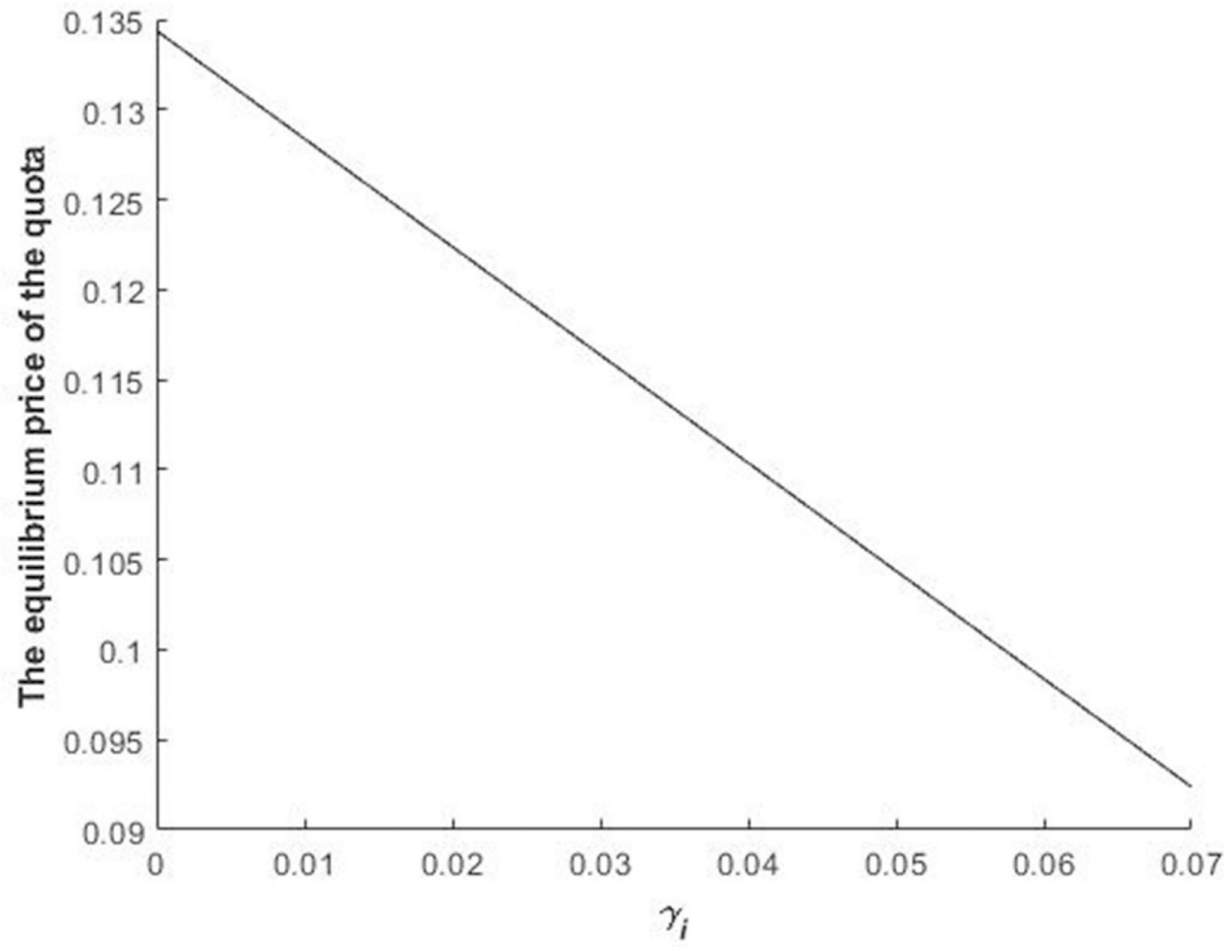
Impact of *γ*_*i*_ on the bidders’ equilibrium bidding price of the quota during the first stage.

Figs 2 and 3 illustrate the impact of *R* and *γ*_*i*_ on the equilibrium bidding price of bidders in the first stage.

The equilibrium bidding price of bidders during the first stage increases with the increase in *R* (Fig 2) because the larger is *R*, the higher are the fine of delaying construction and the construction land quota. Therefore, the equilibrium bidding price of bidders in the first stage increases.

Fig 3 shows that the bidding price of bidders during the first stage decreases with the increase in *γ*_*i*_. As aforementioned, the increase in bidders’ revenue loss results in quota value depreciation. Consequently, bidders decrease their bidding.

**Fig 4:**
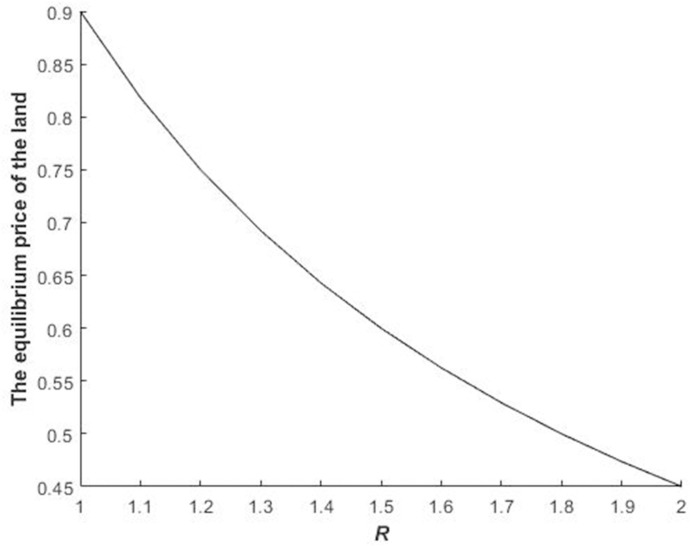
Impact of *R* on equilibrium bidding prices during the second stage for bidders who fail to obtain the quota.

Fig 4 presents the impact of *R* on the equilibrium bidding price in the second stage for the bidders who fail in the first stage.

With the improvement of the fine for delaying construction, the equilibrium bidding price of losers decreases during the second stage (Fig 4). This finding is accurate. The core reason for this phenomenon is that when *R* is high, the additional cost for the loser who obtains the land during the second stage increases in the future. Thus, the loser pays a higher cost if they acquire the land and is willing to pay less for the construction land auction. Fig 4 shows that the parameter of land price depreciation negatively affects the losers’ value of the construction land.

Figs 2–4 illustrate Propositions 2 and 3.

**Fig 5:**
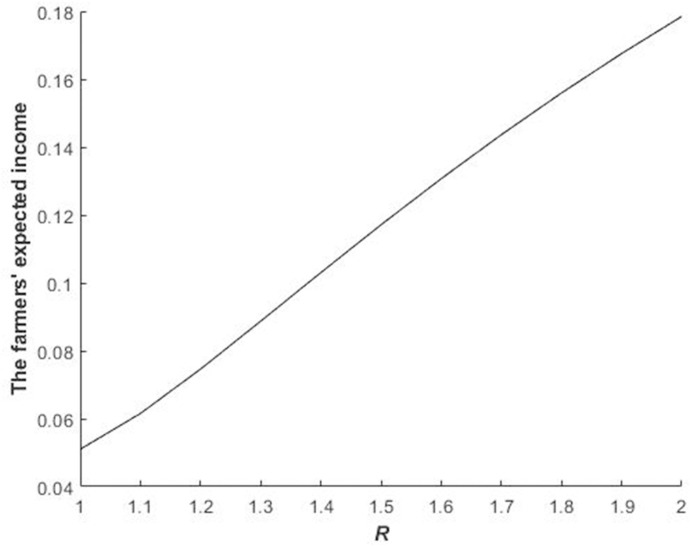
Impact of *R* on the farmers’ expected income.

**Fig 6:**
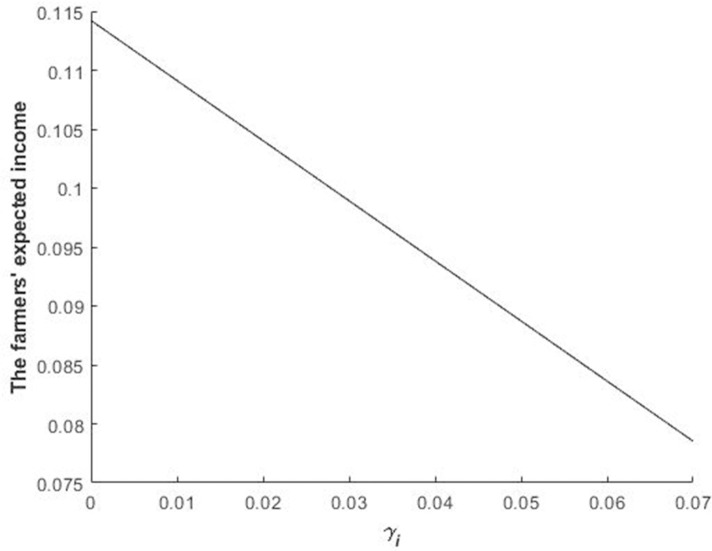
Impact of *γ*_*i*_ on the farmers’ expected income.

We drew a picture to further analyse the impact of *R* and *γ*_*i*_ on the expected income of farmers. Figs 5 and 6 are drawn using the equilibrium conditions and by calculating expressions.

The farmers’ expected income increases with the increase in the fine on delaying construction and decreases with the increase in the revenue loss of bidders losing during the second stage (Figs 5 and 6) because the equilibrium bidding price of the construction land quota increases and decreases with the increase in the fine and revenue loss, respectively, that is, the fine of delaying construction and repurchasing the quota at a premium from the bidder who acquires the quota but fails to obtain the land set by local governments indirectly affects the income of farmers who reclaim idle homesteads. Therefore, the fine on delaying construction can bring higher compensation benefits to farmers and encourage relatively more farmers to reclaim land to improve the utilisation efficiency of rural construction lands.

Farmers who reclaim their idle homesteads are keen to share the process benefits. Their concern is always about receiving compensation. Compensation currently received by farmers is usually small compared with the security the land provides to Chinese farmers. Therefore, compensation for farmers can be indirectly increased by raising the fine of delaying construction, which can further release potential land resources in rural areas. This compensation also promotes the sustainable development of urban and rural areas in China. Therefore, Proposition 6 is verified.

## Discussion

The findings of analysis are as follows: First, our improved two-stage sequential auction, with complementarity, is a reasonable and feasible approach to price the quota as the equilibrium bidding price. The quota reflects its true value for bidders and can effectively encourage farmers. The current reclamation-cost-based pricing (adopted by Chongqing and many other cities, such as Nanjing and Guangzhou) is unreasonable, and hence, cannot to motivate farmers [21, 22].

Second, the equilibrium bidding prices of the quota, or the item in the first stage, rises with the increase in the parameter of land value depreciation *R*. This finding is consistent with the conclusions drawn by Wang et al. [36] and Leufkens and Peeters [45]. The results show that *R* positively affects the quota price. In practice, *R* is positively correlated to the fine for developers delaying construction. The higher is the fine, the higher is the cost saved by the quota, that is, the value and price of the quota increases with the increase in *R*. Moreover, the bidding price of land, or the item during the second stage of bidders who fail in the first stage, decreases with the increase in *R*. This result is indirectly supported by Brendstrup [46]. The political implication of this finding is that governments can raise the fine for developers delaying construction to increase their bidding price of the quota, thereby increasing farmers’ income.

Third, the equilibrium bidding price of the quota decreases with the increase in the number of bidders. This result strongly agreed with that of Sørensen [33], who proved that the bidders bid less aggressively during the first auction when the number of bidders increases. In reality, when many bidders are present in the auction, the competition among them becomes more intense. Thus, the probability of a bidder obtaining the quota, and the relative expectation of the extra revenue, decreases. Hence, bidders pay less for the quota.

Finally, the equilibrium bidding price of the quota decreases and increases with the increase in the revenue loss and probability, respectively. This finding is consistent with the conclusions drawn by Lu [47]. Local governments should repurchase the quota at a premium from the bidder to increase the bidding price on the quota.

We designed a mechanism to price the quota and land and used Chongqing and Chengdu as the reference. Therefore, our model and conclusions can be applied to the allocation and use of resources with constraints. First, the transaction system of the quota is widely adopted in numerous cities in China. In the European Union, the use of railways is split into several segments and periods, because their use is complementary [38]. Our model and conclusions are applicable to governments and decision-makers for their practices.

## Conclusion

This study improved the two-stage sequential auction model of complementary items and used this enhanced model to study pricing mechanism of the quota. Through theoretical and numerical analyses, we obtained the optimal bidding price on the quota and land and the impacts of relative factors on the quota bidding price. Compared with the current reclamation-cost-based price mechanism, our mechanism is reasonable and feasible because it can reveal the true value of the quota for bidders and can effectively stimulate farmers’ motivations of reclaiming their idle homesteads. The equilibrium bidding price on the quota decreases with the increase in the number of bidders or bidders’ revenue losses caused by losing land. The equilibrium bidding price on the quota increases with the increase in the fine of delaying construction or the probability of bidders’ belief of winning the land. Therefore, local governments can appropriately increase the fine for delaying construction and/or repurchase the quota at a premium from the bidder who fails to obtain the land to raise the value and price of the quota. Consequently, more farmers are motivated to reclaim their idle homesteads to generate abundant quotas. As a result, the efficiency of land use and urbanisation in China can be improved.

Although our model provides a novel method for exploring the intrinsic value of construction land quotas and solving the problem between the supply and demand of construction lands, further examinations should be conducted. First, we considered that there is no threshold for entering the second auction. Chengdu implemented the rule of owning the quota as the threshold. Its price was considerably higher than that of the quota in Chongqing, where the threshold was not implemented [48, 49]. Therefore, studying the pricing mechanism with the rule of threshold and discussing its impacts on the prices of the quota and land is valuable. Second, this paper does not consider that the government can provide bidders a discount on the construction land, according to their price of the quota, which is applied in Chongqing. Therefore, studying whether and how the price discount affects the prices of the quota and land is useful.
